# High throughput long-read sequencing of circulating lymphocytes of the evolutionarily distant sea lamprey reveals diversity and common elements of the variable lymphocyte receptor B (VLRB) repertoire

**DOI:** 10.3389/fimmu.2024.1427075

**Published:** 2024-08-07

**Authors:** Amin Zia, Ariel Orozco, Irene S. Y. Fang, Aspen M. Tang, Ana Sofia Mendoza Viruega, Shilan Dong, Leslie Y. T. Leung, Vijaya M. Devraj, Opeyemi E. Oludada, Götz R. A. Ehrhardt

**Affiliations:** ^1^ dYcode, Toronto, ON, Canada; ^2^ Department of Immunology, University of Toronto, Toronto, ON, Canada

**Keywords:** variable lymphocyte receptor, jawless vertebrates, immune repertoire, antibodies, longread sequencing

## Abstract

The leucine-rich repeat-based variable lymphocyte receptor B (VLRB) antibody system of jawless vertebrates is capable of generating an antibody repertoire equal to or exceeding the diversity of antibody repertoires of jawed vertebrates. Unlike immunoglobulin-based immune repertoires, the VLRB repertoire diversity is characterized by variable lengths of VLRB encoding transcripts, rendering conventional immunoreceptor repertoire sequencing approaches unsuitable for VLRB repertoire sequencing. Here we demonstrate that long-read single-molecule real-time (SMRT) sequencing (PacBio) approaches permit the efficient large-scale assessment of the VLRB repertoire. We present a computational pipeline for sequence data processing and provide the first repertoire-based analysis of VLRB protein characteristics including properties of its subunits and regions of diversity within each structural leucine-rich repeat subunit. Our study provides a template to explore changes in the VLRB repertoire during immune responses and to establish large scale VLRB repertoire databases for computational approaches aimed at isolating monoclonal VLRB reagents for biomedical research and clinical applications.

## Introduction

Antibodies are instrumental in providing long lasting humoral protection to pathogenic challenges, both in the context of natural infection and vaccination. The vast immunoglobulin-based antibody repertoire is generated by combinatorial diversity of the variable (V), diversity (D), and joining (J) gene segments and V, J gene segments of the heavy and light chain gene loci, respectively (1, 2). This repertoire can be further diversified by the incorporation of somatic mutations during affinity maturation, a process occurring in the specialized microenvironment of germinal centers (GC) that leads to the generation of high affinity antibodies (3). While the amplification of heavy and light chain gene sequences by PCR from individual cells provided a wealth of information on antibody characteristics for specific antigens response (4–9), only large-scale immune receptor sequencing studies, either on bulk populations or on single cell level, provided insights into the complexity of B cell responses, differences of antigen receptors encoded by distinct B cell subpopulations in circulating and tissue-bound cells, and the clonal relationships of B lineage cells during the course of an immune response (10–13). Extensive antigen receptor sequence databases also form the backbone for machine learning strategies geared towards *in silico* generation of monoclonal antibodies with defined antigen binding characteristics (14–16).

**Figure 1:**
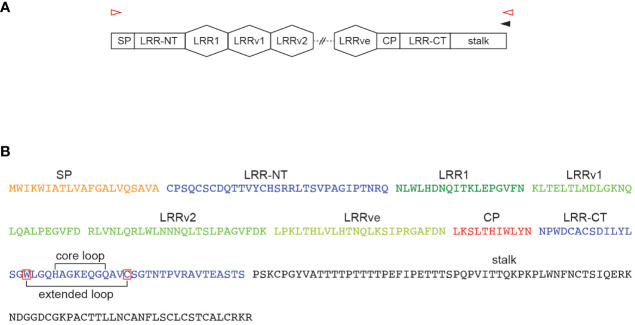
Structural components of VLRB antibodies. **(A)** Structural elements of a mature VLRB coding sequences composed of signal peptide (SP), capping N-terminal LRR (LRR-NT), LRR1, 0-9 variable LRRv, a single LRRve module, connecting peptide (CP), capping C-terminal LRR (LRR-CT) and invariant stalk region. Approximate positions of oligonucleotides for the reverse transcription reaction (black arrowhead) and cDNA amplification (red arrowheads) are indicated. **(B)** Protein sequence of a representative VLRB molecule with 2 LRRv units. Sequences within the LRR-CT corresponding to the core loop and extended loop are indicated by brackets. Conserved tryptophan (in -4 position of the N-terminus of the core loop) and cysteine (in +3 position of the C-terminus of the core loop) residues defining the extended loop are indicated by red frames.

Unlike conventional antibodies which are based on the immunoglobulin fold, the non-conventional variable lymphocyte receptor (VLR) antibody system of the evolutionarily distant jawless vertebrates utilizes the β-sheet forming leucine-rich repeat (LRR) as basic structural unit (17, 18). The last common ancestor of jawless and jawed vertebrates occurred approximately 500 million years ago with lampreys and hagfish being the only extant members of this group. Studies of the adaptive immune system of jawless vertebrates that followed the initial discovery of the somatically diversifying VLR antibody system (17) revealed numerous remarkably conserved features alongside the structurally distinct antigen receptors (19). Three VLR genes, *VLRA*, *VLRB*, and *VLRC*, have been identified which are expressed in a mutually exclusive pattern of expression on cells with gene signatures resembling those of mammalian αβ T cells, B cells, and γδ T cells, respectively, but only VLRB molecules are expressed on the cell surface and are secreted as disulfide-linked multimers (20–22). In addition, a recent study identified VLRD and VLRE somatically diversifying receptor systems that are phylogenetically related to the VLRA and VLRC genes (23). The potential VLRB repertoire is predicted to exceed 10^14^ clonotypes. A single incomplete VLRB gene is flanked by numerous diverse LRR cassette sequences and a gene conversion-like mechanism leads to the generation of a mature VLRB gene consisting of a signal peptide (SP), capping N-terminal LRR, a conserved LRR1, up to 9 variable LRRv cassettes, connecting peptide (CP), capping C-terminal LRR and an invariable stalk region containing multiple C-terminal cysteine residues involved in the formation of disulfide-linked, soluble decamers (**Figure 1**) (24, 25). In contrast to the idiotype of conventional antibodies which is formed by residues encoded by matching heavy and light chain subunits, secreted VLRB antibodies are generated by iteration of a single VLRB polypeptide (20). Exceptions to the requirement of matching heavy and light chain sequences are represented by single chain antibodies found in camelids and cartilaginous fish (26). Structural analyses of monoclonal VLRB antibodies in complex with protein or carbohydrate antigens revealed stark differences in VLRB antibody-antigen binding between VLRB antibodies and conventional antibodies. VLRB antibodies assume a solenoid shape with antigen-interacting residues positioned in the inner concave surface and in a variable, flexible loop structure protruding from the capping C-terminal LRR (27, 28). The unique structural characteristics of VLRB antibodies prompted the exploration of their suitability as novel biomarker discovery reagents and resulted in the isolation of monoclonal VLRB antibodies with unique recognition of memory B cells, plasma cells, blood-brain barrier epithelia, or sulfated carbohydrate antigens (29–33).

In contrast to conventional antibodies where diversification mechanisms occur without extensive changes affecting overall length of the heavy and light chain coding sequences, a key factor contributing to VLR antibody diversity is the number of distinct LRRv segments incorporated into the mature protein, each of which is typically encoded by a 24-amino acid (aa)/72-nucleotide (nt) cassette. The resulting size diversity of VLRB encoding sequences makes standard low-throughput approaches, such as single-cell Sanger sequencing, of immune receptor sequencing approaches unsuitable for repertoire analyses of jawless vertebrates. In this study, we explored the feasibility of high-throughput sequence analysis of the sea lamprey VLRB immune repertoire. We present a protocol for the use of PacBio sequencing and characterization of mature full-length VLRB antibodies and provide insights into previously unrecognized characteristics of VLRB antibodies encoded by circulating sea lamprey larvae lymphocytes.

## Materials and methods

### Sea lamprey larvae lymphocyte isolation and RNA extraction

Sea lamprey *(Petromyzon marinus)* larvae 10-12 cm in length and approximately 2-4 years of age were obtained from the United States Geological Survey (Great Lakes Science Center, Millersburg, MI). Six animals were humanely euthanized by immersion in MS-222 (Sigma-Aldrich, St. Louis, MO) and circulating lamprey lymphocytes were purified by density gradient centrifugation using 50% Percoll. Lamprey lymphocytes retained at the interphase of the Percoll layer were collected, washed twice with 0.66 x PBS and subjected to total RNA purification using RNeasy spin columns (Qiagen, Hilden, Germany). Sea lamprey experiments were approved by the animal care committee of the University of Toronto.

### Amplification of VLRB sequences and library generation

1^st^ strand cDNA reactions were performed on 750 ng of total RNA separately from each animal using the Superscript IV reverse transcriptase (Invitrogen, Waltham, MA). The oligonucleotide used for reverse transcription (5’ – *TATTTCCAGCACACTGGATCAGNNNNNNNNNNNNTCAACGTTTCCTGCAGAGGGCG* – 3’) contained sequences specifically annealing in the invariant stalk region of VLRB transcripts (shown in bold) (**Figure 1A**), a 12 nt unique molecular identifier (UMI, indicated by N) and the annealing sequence for the PCR antisense oligonucleotide (underlined). Each cDNA reaction was split into 8 aliquots for subsequent library amplification using KOD Hotstart DNA Polymerase (Millipore-Sigma, Burlington, MA) for 25 cycles at 57°C. Forward (5’ – *TATNNNNNNgtggatcaagtggatcgc* – 3’) and reverse (5’ – *TATNNNNNNCCAGCACACTGGATCAG* – 3’) oligonucleotides for PCR amplification contained sequences specific for the invariant SP of VLRB and the reverse transcription oligonucleotide, respectively, as well as a 6 nt sample identifier (indicated by N). The aliquots from each library were pooled, purified using NucleoSpin gel and PCR clean-up columns (Machery-Nagel, Düren, Germany) and amplicon quality was verified by DNA gel electrophoresis (**Supplementary Figure 1**). The independently generated libraries without shearing and size selection were pooled and loaded to a single PacBio 8M Sequel-II SMRT cell. PacBio sequencing was performed by the Centre for Applied Genomics, The Hospital for Sick Children, Toronto, ON, Canada. Data for the sequenced samples were deposited at the Sequence Read Archive (SRA) with accession number SRR29656293.

### Sequence data processing

**Figure 2:**
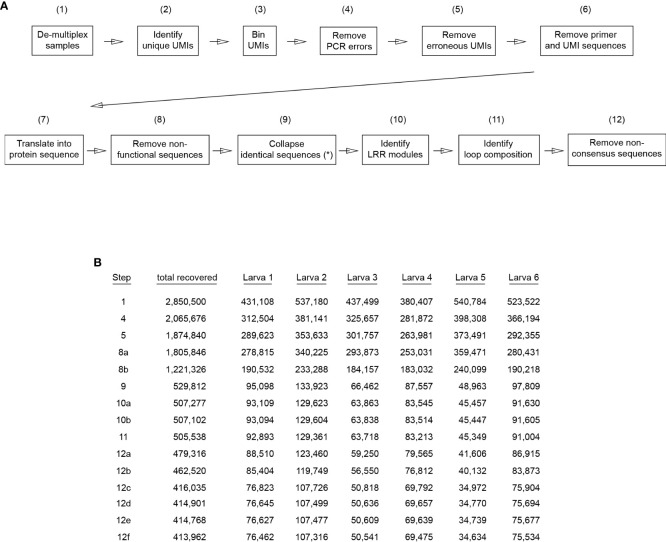
Workflow for VLRB library analysis. **(A)** Sequences from each of the generated libraries were analyzed separately. Numbers in brackets indicate the VLRB sequences remaining after each analysis step (combined libraries). (*) Identical sequences were collapsed independently for each larva dataset. **(B)** Stepwise reduction of VLRB sequences during sequence analysis for each individual larva dataset. Listed steps correspond to numbers in brackets in **(A)** and are shown for those that result in exclusion of VLRB sequences. Steps 8a and 8b indicate premature stop codons and absence of stalk sequences, respectively. Steps 10a and 10b indicate absence of LRR1 and absence of LRRV modules, respectively. Steps 12a-f indicate removal of VLRB sequences with LRRv modules different from 24 aa length, LRR1 modules different from 18 aa length, LRR-NT modules different from 31 aa length, stalk region different from 87 aa length, SP different from 21 aa length and CP different from 11 aa length, respectively.

Raw sequencing data was processed by PacBio’s on-instrument Circular Consensus Sequencing (CCS) workflow, resulting in 3,199,280 (53.2%) HiFi reads with quality value (QV ≥ 20) and 2,818,567 (46.8%) non-HiFi sequencing reads. Only HiFi reads were included in this study. The CCS workflow generates the HiFi reads with 99.9% accuracy (34). A graphic flow chart delineating the sequence data processing steps and statistics of recovered data in each step are shown in **Figure 2**. Initially, the read DNA sequences were trimmed using the PCR primer sets selected to generate the amplicons and were demultiplexed for each individual sea larva sample by using the 6nt sample-specific barcodes, resulting in a total of 2,850,500 DNA sequences. DNA sequences lacking perfect PCR primers or sample-specific barcodes were discarded. For each individual animal sample set, the 12-bp UMIs were binned and reads with identical UMIs and VLR DNA sequences with ≥ 99% nucleotide identity were clustered and collapsed using USEARCH (35) to remove PCR amplification bias and errors. The collapsed DNA sequences represent UMI-barcoded RNA molecules that accumulated PCR mutations fewer than 1% of VLR length during amplification and sequencing. DNA sequences with identical UMI and more than 1% VLRB nucleotide variation were discarded at this stage, resulting in a total of 2,065,676 DNA sequences. Next, identical VLR DNA sequences with different UMIs within one nt (out of 12 nt total UMI length), were identified and removed from dataset since these UMIs (15,421) most likely accumulated a single erroneous mutation during PCR amplification. Finally, the oligonucleotide primers and UMI sequences were removed from reads, resulting in 1,874,840 DNA sequences, each of which correspond to a single RNA sequence in the primary library.

The nucleotide DNA sequences were translated into amino acid residues starting at the N-terminus, and sequences with a premature stop codon were removed from analysis (68,994). Next, protein sequences that did not carry or were within 3 or more residue differences of the invariant VLRB C-terminal stalk region with motif “DCGKPACTTLLNCANFLSCLCSTCALCRKR” were removed from further analysis. This resulted in a total of 1,221,326 protein sequences. Lastly, for each animal sample dataset, all identical protein sequences (with an average 2.87 +/-0.96 SD identical protein sequences per sample) were collapsed, resulting in a total of 529,812 unique VLRB protein sequences, which were used for further analysis.

### LRR1 subunit detection

The LRR1 subunit consists of 5 conserved amino acid residues with the motif “xLxLxxNxxxxLxxxxFx”. We used a 30-residue sliding window to scan VLRs from the end of SP to the CP, and at each step, we aligned the 24 aa resulting peptide with the LRR1 motif using ClustalW (36). We detected LRR1 if the alignment did not result in identical residues with the 5 conserved aa in the motif or the alignment results in more than 6 gaps in the motif. Protein sequences in which no LRR1 was detected were discarded, resulting in a total of 507,227 VLR protein sequences.

### LRRV subunit detection

Once LRR1 subunit were detected, we scanned the VLR from the end of LRR1 to CP and searched for any peptide sequence with minimum length of 15 aa that aligned with the LRRv motif “xLxxLxxLxLx” requiring an exact match with conserved leucine residues in the motif and a maximum of 10 alignment gaps with the motif. Protein sequences with no detected LRRV were discarded, resulting in a total of 507, 102 VLR protein sequences.

### LRR-CT loop detection

The protein sequence region between CP to stalk of each VLR were aligned to a randomly selected set of 50 VLRs that were used as template, and loops were identified as the stretch of amino acid residues starting at position +4 of the upstream conserved W and ended at the position -3 of the downstream conserved C (Wxxx-loop-xxC). Here, we refer to the loop and the 7 aa flanking residues as extended loop and to the loop without the 7 aa flanking residues as core loop. Protein sequences without a detected loop were removed from the analysis, resulting in a total of 505,538 VLR protein sequences.

### Filtering of erroneous sequences

Analysis of VLRB subunits revealed a small number of sequences with inconsistent subunit length. We therefore sequentially removed 26,222 sequences with LRRv length other than 24 aa, 16,796 sequences with LRR1 length other than 18 aa, 46,485 sequences with LRR-NT module length other than 31 aa, 1,134 sequences with stalk length other than 87 aa, 133 sequences with SP length other than 21 aa, and 806 sequences with CP length other than 11 aa from the final datasets. This resulted in a combined 413,962 VLR sequences from six animals.

### Clustering analysis and Amino-acid properties determination

Clustering analysis were performed using a locally installed version of CDHIT (V4.8.1) (37, 38) with default parameters and with selected similarity thresholds. The CDHIT outputs were parsed and analyzed using customized scripts. The cumulative distribution function of cluster sizes was used as surrogate measure to compare diversity of sequences. Alikazam (39) was used to compute amino acid properties of protein sequences.

## Results

### Sea lamprey larvae display a highly diverse VLRB repertoire among circulating lymphocytes

**Figure 3:**
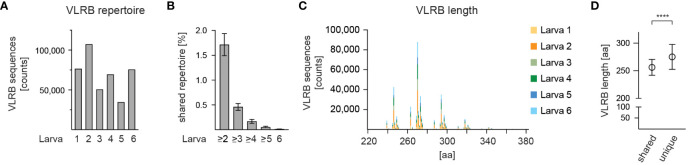
Frequency and size of circulating VLRB molecules. **(A)** Numbers of mature VLRB sequences isolated from 6 individual larvae. **(B)** Frequency of shared VLRB sequences with 100% sequence identity detected between the indicated number of larvae. Bars indicate mean +/- SD. **(C)** Length distribution of mature VLRB sequences. Depicted are absolute numbers of isolated VLRB sequences. Numbers indicate amino acids, including those encoding the SP. Sequences longer than 380 aa representing <0.06% of total sequences were omitted. **(D)** Comparison of VLRB length of shared vs unique VLRB sequences. Symbols represent mean +/- SD. Statistical significance was determined using an unpaired *t*-test and is indicated by asterisks: (****) p<0.0001.

We explored the extended length of sequencing reads provided by the PacBio sequencing platform to assess the complexity of the VLRB antibody repertoire of jawless sea lamprey larvae. Total RNA obtained from circulating lymphocytes of six sea lamprey larvae was reverse transcribed and amplified using VLRB-specific oligonucleotides (see **Figures 1A, B**) that embedded unique molecular barcodes (UMIs) and sample-specific barcodes and was multiplexed into a pooled library with the majority of DNA fragments ranging from 800-1,100 bp (**Supplementary Figure 1**). The pooled library was sequenced on a PacBio Sequel-II SMRT Cell platform, resulting in a high-throughput long-read sequencing dataset consisting of a total of 3,199,280 HiFi sequencing reads. The sequencing reads were pre-processed using an in-house computational pipeline (**Figure 2**) where they were trimmed of adapters, demultiplexed for each animal sample, and corrected for PCR errors and duplication biases using UMIs. The resulting nucleotide sequences were translated into protein sequences and any protein sequence that contained a premature stop codon or lacked the conserved C-terminal stalk region were removed from further analysis. The final dataset consisted of a total of 413,962 unique VLRB sequences ranging from 34,634 to 107,316 sequences from each of six animals (**Figure 3A**). Cluster analysis showed several extended clusters in each of the larval data sets (**Supplementary Figure 2**). Furthermore, clustering of VLRB sequences based on 100% sequence identity revealed that 7,078 (1.71%), 1,904 (0.46%), 704 (0.17%), 207 (0.05%), and 41 (0.01%) of the sequences were shared between at least 2, 3, 4, 5, and 6 animals, respectively (**Figure 3B**). The overall length VLRB protein sequences including invariant stalk region ranged from 238 to 438 aa that grouped in multiples of approximate 24 aa, consistent with a variable number of incorporated LRRv modules (**Figure 3C** and **Supplementary Figure 2**). Perhaps unsurprisingly, VLRB sequences that were shared among two or more larvae were shorter compared to unique VLRB sequences (**Figure 3D**).

Recent work by Das et al. provided a detailed analysis of the genomic *VLRB* gene locus (40). *S*equences encoding the SP linked to 5’ sequences of the LRR-NT and the invariant stalk region linked to 3’ sequences of the LRR-CT originate from the single, *VLRB* germline gene and are not subject to somatic diversification. To account for any residual sequencing error or inherent noise floor we started with the VLRB stalk subunit, encoded by a single genomic locus. We clustered all VLRB stalk sequences with 100% sequence identity and relaxed the clustering by stepwise reducing the clustering threshold. We observed that at a clustering with 95% sequence similarity, virtually all invariant stalk transcripts were covered in a single cluster (**Table 1**). Similar analysis for SP sequences yielded comparable results (**Table 1**). We therefore used 95% sequence similarity as a reference threshold for clustering of sequences encoding distinct structural VLRB elements and sequence diversity assessment. However, it should be noted that under these conditions the number of observed clusters of VLRB components that are subject to somatic diversification outnumbered the described genomic loci, indicative of additional diversification mechanisms such as the use of partial LRRv cassette templates in the assembly process (40).

**Table 1:** Determination of inherent sequencing errors.

Subunit	SP	LRR-NT	LRR1	LRRv	CP	LRR-CT	loop	stalk
Number of genomic loci (N)	1	79	66	601	29	91	91	1
% subunits in N clusters (total clusters) (65% aa similarity clustering)	100 (2)	100 (30)	100 (30)	100 (178)	100 (14)	100 (26)	100 (77)	100 (1)
% subunits in N clusters (total clusters) (70% aa similarity clustering)	100 (2)	100 (46)	100 (46)	100 (291)	100 (14)	100 (38)	100 (84)	100 (5)
% subunits in N clusters (total clusters) (75% aa similarity clustering)	100 (2)	100 (75)	99.99 (75)	100 (552)	100 (24)	100 (53)	99.99 (95)	100 (5)
% subunits in N clusters (total clusters) (85% aa similarity clustering)	100 (2)	99.60 (219)	99.30 (219)	94.41 (4,085)	99.33 (123)	100 (88)	99.81 (241)	100 (5)
% subunits in N clusters (total clusters) (95% aa similarity clustering)	100 (5)	88.88 (2,393)	87.42 (2,394)	79.89 (16,638)	96.35 (352)	99.55 (333)	98.84 (793)	99.96 (5)
% subunits in N clusters (total clusters) (100% aa similarity clustering)	99.47 (68)	79.89 (4,912)	78.03 (4,912)	69.89 (29,501)	96.35 (352)	94.34 (2,535)	98.84 (849)	94.98 (521)

Cluster formation of sequences corresponding to the various VLRB subunits. Numbers of genomic loci are based on published reports by Das et al. (40). Cluster analysis of VLRB sequences from 6 combined libraries was performed with 100% amino acid similarity and stepwise relaxation of similarity thresholds to 65%. A 95% similarity threshold was selected for subsequent sequence analyses.

**Figure 4:**
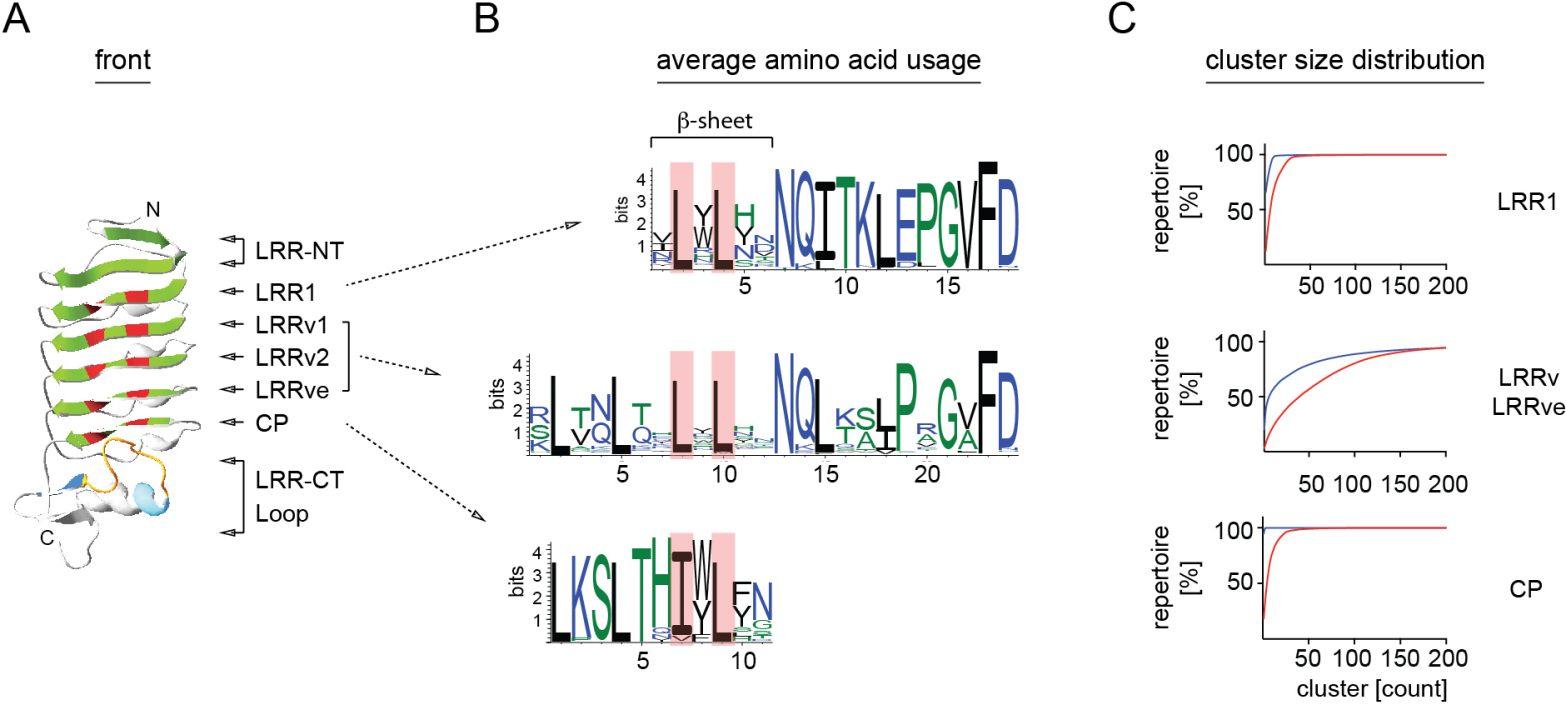
Amino acid diversity of VLRB LRR1, LRRv and CP modules. **(A)** Ribbon model of a VLRB antigen binding domain. Parallel β-sheets are shown in green and conserved leucine/isoleucine residues forming the hydrophobic core of the molecule are depicted in red. Core and extended loop sequences of the LRR-CT are shown in orange and blue, respectively. Protein model was generated using the Phyre2 modeling portal (42). **(B)** Amino acid usage of LRR1, LRRv and CP sequences. Conserved leucine/isoleucine residues are indicated by red background. Illustration was generated using the WebLogo tool (43). **(C)** Cluster size cumulative distribution of LRR1, LRRv/LRRve and CP segments. Cluster sizes were calculated separately for 85% similarity for sequences involved in β-sheet formation as indicated in **(B)** (red) or not (blue) and are shown in order ranging from largest to smallest clusters.

Analysis of LRR1 and LRRv modules confirmed the initial observations by Pancer et al. (17) based on a limited number VLRB sequences that LRR1 modules are predominantly 18 aa in length and LRRv modules 24 aa in length. For a detailed analysis of LRR element usage in the VLRB repertoire, we set stringent parameters for a consensus VLRB sequence based on previously established VLRB structural elements (**Figure 1A** and **Table 1**) (17, 24, 41). These required that the capping LRR-NT was followed by an 18 aa LRR1 module which in turn was followed at least one 24 aa LRRv unit. Next, an 11 aa CP bridged the LRRve unit with the LRR-CT. Sequences that did not match all structural elements were excluded. As expected, the highest degree of diversity was observed in the LRRv and LRRve modules compared to the more conserved LRR1 modules. Within each module, sequences encoding the β-sheets displayed highly conserved leucine residues flanked by highly diverse residues that form (a part of) the antigen interacting region (**Figures 4A–C**).

**Figure 5:**
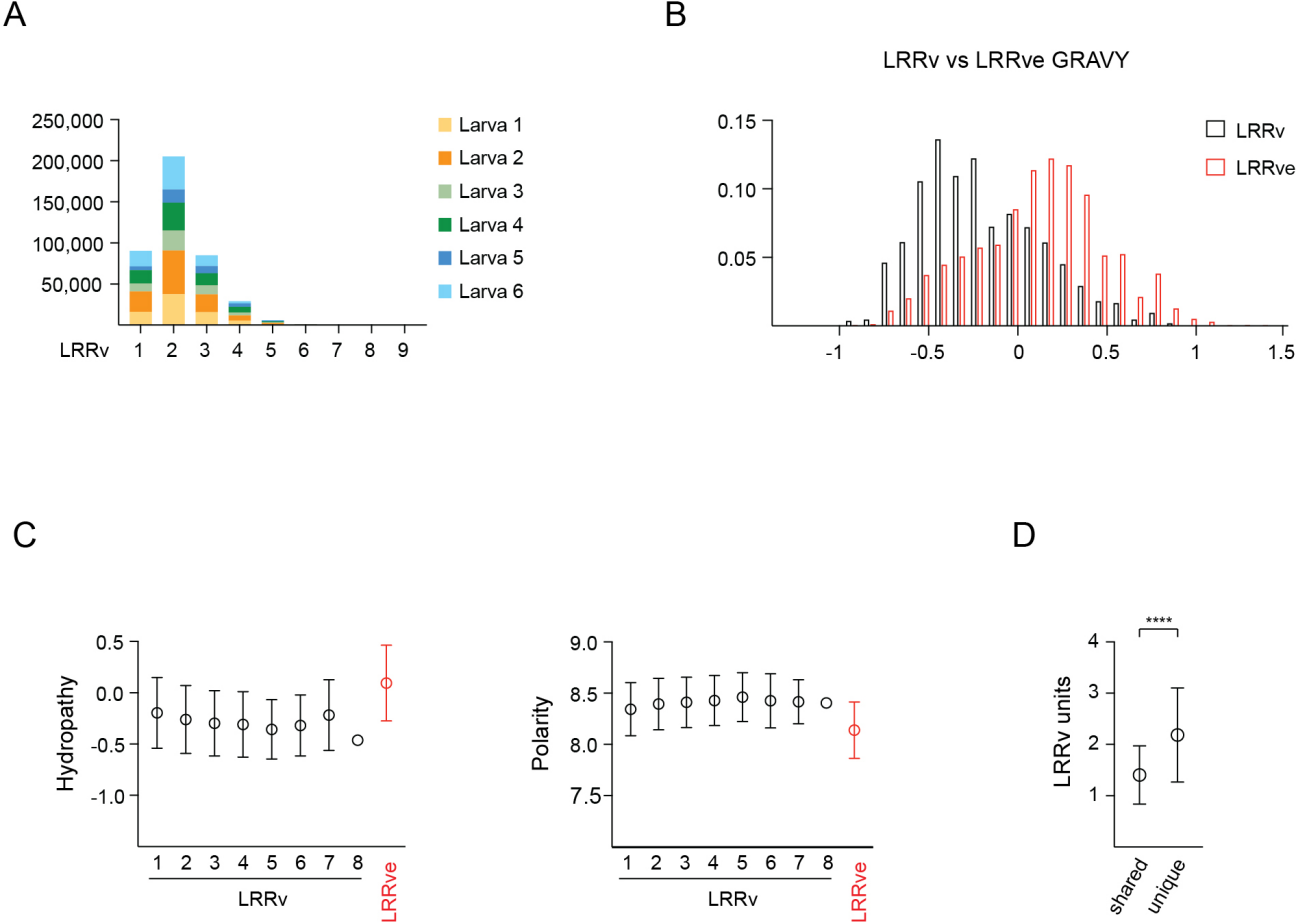
LRRv composition of mature VLRB sequences. **(A)** Numbers of LRRv elements incorporated into mature VLRB sequences. VLRB molecules with a single with one LRRv unit contain only the LRRve segment. **(B)** Hydrophathy analyses of LRRv and LRRve units. Bars represent mean +/- SD for LRRv and LRRve. **(C)** Positional hydrophathy (left) and polarity (right) analyses of LRRv and LRRve units. Numbers indicate the LRRv position and values are depicted as mean +/- SD. **(D)** Comparison of LRRv units incorporated into shared vs unique VLRB sequences. Symbols represent mean +/- SD. Statistical significance was determined using an unpaired *t*-test and is indicated by asterisks: (****) p<0.0001.

Detailed analysis of the LRRv/LRRve units incorporated into each VLRB molecule revealed that VLRB proteins composed of two LRRv units were detected most frequently with an average of 2.17 LRRv modules per molecule (**Figure 5A** and **Supplementary Figure 2**). A comparison of hydropathy values for LRRv versus LRRve modules revealed that LRRv modules were more hydrophilic than LRRve modules (**Figure 5B**). Hydrophobicity of individual LRRv modules decreased from N-terminal to C-terminal location and greatly increased for the most C-terminally located LRRve (**Figure 5C**, left panel). Conversely, amino acid polarity scores increased for LRRv modules from N-terminal to C-terminal orientation but were markedly lower for LRRve modules (**Figure 5C**, right panel). As expected, based on observations of the overall length of VLRB sequences shared among two or more larvae, shared VLRB sequences incorporated fewer LRRv units compared to unique VLRB sequences (**Figure 5D**).

**Figure 6:**
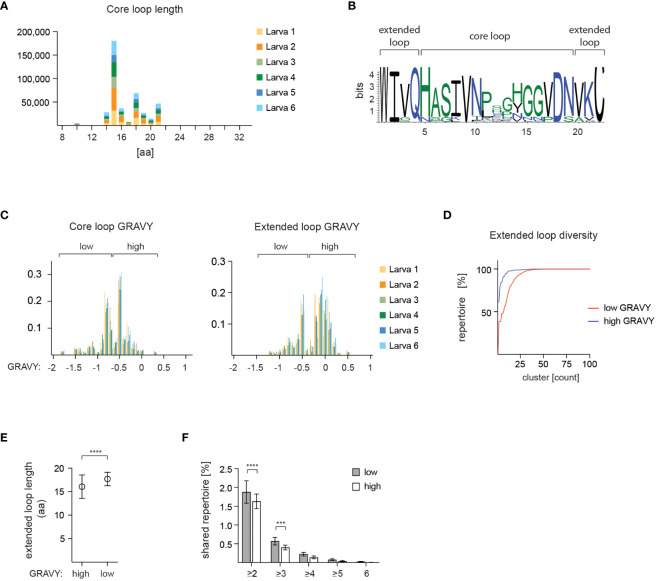
Characteristics of VLRB C-terminal loop sequences. **(A)** Core loop sequences for 6 experimental larvae are depicted in amino acids (aa) numbers. **(B)** Amino acid usage of core and extended loop sequences depicted for the most frequently observed loop length of 15 aa (core loop)/22 aa (extended loop). **(C)** Hydropathy scores shown for 6 experimental larvae. Loop sequences are grouped in 0.1 score increments and each group is normalized to the total number of loop sequences for each larva. The threshold to separate high and low hydropathy value groups for extended loop analyses was set at 0.35. **(D)** Cluster size distribution of high (blue) and low (red) hydropathy extended loop sequences. Cluster sizes were calculated based on 85% similarity. **(E)** Calculation of extended loop length for sequences with high or low hydropathy scores. Symbols indicate mean +/- SD, statistical significance was determined using an unpaired *t* test and is indicated by asterisks. **(F)** Distribution of shared VLRB sequences between at least 2, 3, 4, 5, or 6 larvae, respectively, with high and low hydropathy scores. Bars indicate mean +/- SD, statistical significance was determined with a 2-way ANOVA and Sidak’s posthoc multiple comparison test and is indicated by asterisks: (***) p<0.001, (****) p<0.0001.

A prominent feature of VLRB molecules is a variable loop protruding from the LRR-CT module that is frequently involved in antigen binding (27, 28, 44). Structural analyses of VLRB antibodies indicate that residues composing this loop are preceded by a conserved tryptophan in the -4 position and followed by a conserved cysteine in the +3 position (**Figure 1B**). Here we refer to the sequences up to and including flanking residues as ‘extended loop’ whereas loop sequences without flanking residues are referred to as ‘core loop’. The most frequent core loop length was 15 aa with an overall mean core loop length of 16.6 +/- 2.3 aa (**Figure 6A** and **Supplementary Figure 2**). Sequence variability was mostly restricted to the core loop region whereas the flanking residues contained in the extended loop region displayed a higher degree of sequence conservation (**Figure 6B**). Calculation of the hydropathy values for the loop sequences revealed a bimodal pattern that was observed for core loop and extended loop sequences (**Figure 6C**), although it was more pronounced in the extended loop data set, indicating that the residues flanking the core loop enhanced the distinct hydropathy characteristics. We then explored potential differences in loop diversity based on hydropathy scores. This analysis showed that the loop repertoire with low hydropathy scores was more diverse compared to the repertoire characterized by high hydropathy values (**Figure 6D**). Interestingly, we also noted that loop sequences with lower hydropathy values were longer than loop sequences with higher hydropathy scores (**Figure 6E**) and mature VLRB sequences harboring low hydropathy loop scores were more frequently shared between the analyzed larvae (**Figure 6F**).

## Discussion

In this study we present evidence of large-scale antigen receptor data generation of the non-conventional VLRB anticipatory receptor system of the evolutionarily distant sea lamprey. The V(D)J gene segment-based diversification system of conventional antibodies of jawed vertebrates results in heavy and light chain coding sequence that are diverse in composition but similar in length. In contrast, the diverse VLRB repertoire of jawless vertebrates is generated not only by the sequence identity of LRR modules but also by the number of incorporated LRRv modules. While we determined that the average number of LRRv modules was 2.18, we observed VLRB sequences with as little as only the single LRRve module and as many as 8 LRRv cassettes in addition to the LRRve module, resulting in size differences of the amplicons exceeding 600 bp. This diversity in amplicon sizes renders conventional immunoreceptor sequencing approaches unsuitable; however, we could demonstrate the suitability of PacBio long-read sequencing to obtain high quality sequences for VLRB repertoire analysis. The ability to obtain large VLRB sequence databases also allowed us to explore differences in amino acid composition of VLRB proteins with diverse numbers of incorporated LRRv units. It is noteworthy that LRRve units consistently displayed the highest hydropathy and lowest polarity values. VLRB molecules with up to 5 LRRv units in addition to the LRRve segment incorporated into the molecule, which includes nearly the entire analyzed repertoire, displayed a continuous decrease in hydropathy and increase polarity with each C-terminally added LRR segment which then precipitously increased in case of hydropathy or decreased in case of polarity for the LRRve unit. While the purpose underpinning this apparent requirement remains to be elucidated it may represent a factor potentially limiting the VLRB repertoire.

For our VLRB repertoire analysis we followed a very stringent approach in that we excluded all sequences that did not match a consensus VLRB molecule; we thus excluded all sequences that contained LRR1 modules that were not 18 aa in length or sequences with LRRv modules with a length distinct from 24 aa (see **Supplementary Table 1**). Nonetheless, it is possible that these VLRB molecules contribute to the functional repertoire even though they differ from the VLRB consensus sequence. For examples, structural analyses of the O13 monoclonal VLRB antibody in complex with the blood group O H-trisaccharide contains one LRRv module with a length of 23 amino acids (45). In addition to the exclusion of VLRB sequences harbored in any tissue resident cells, our conservative analysis of 413,962 consensus sequences obtained from circulating lymphocytes of 6 larvae is therefore presumably not a complete representation of the VLRB repertoire of these animals but may come close to the representation of the blood VLRB repertoire.

We determined a 95% sequence similarity threshold to account for residual sequencing errors and inherent noise of our VLRB sequence database, based on clustering results of sequence elements that are not subject to diversification, i.e. sequences encoding the SP and the invariant stalk region. However, a 95% sequence similarity threshold would predict that the number of loci for different VLRB structural components, particularly for the LRRv modules, would far exceed the number of loci determined in a detailed analysis of the sea lamprey VLRB genome (40). Non-templated immunoreceptor sequence diversification mechanisms such as n-nucleotide addition or mutations as a result of antibody affinity maturation are not described in jawless vertebrates (18). The unexpected diversity of LRRv modules we observed in fully assembled VLRB sequences is therefore most likely the result of (i) the utilization of partial LRRv template cassette sequences during VLRB assembly (40) and (ii) the use of multiple, distinct sites for LRR module priming (41).

A noticeable proportion of the VLRB sequences we obtained contained either premature stop codons or were lacking the invariant stalk region, indicative of frame shifts within the sequence. While precise positive and negative selection mechanisms in jawless vertebrates remain to be elucidated, non-functional VLRB sequences would be expected to occur only at very low frequencies. These non-functional sequences originate most likely from immature lymphocytes co-purified from the typhlosole, a hematopoietic tissue of sea lamprey larvae (46), since our cell isolation approach did not categorically exclude this possibility. Alternatively, these non-functional sequences could be inherent to SMRT sequencing and may require further improvements to be recognized by our pre-processing algorithm.

A variable loop protruding from the otherwise conserved LRR-CT gained attention owing to its frequent involvement in antigen recognition (27, 28, 45). To identify the loop, we determined conserved tryptophan and cysteine residues 4 amino acids N-terminal and 3 amino acids terminal of the loop, respectively, and analyzed amino acid characteristics of the loop sequences with (extended loop) or without (core loop) these flanking sequences. Unexpectedly, we observed a bimodal hydropathy distribution for the extended and core loop and noted that VLRB transcripts with low hydropathy extended loop sequences were more frequently shared among individual larvae. It will be interesting to determine if certain classes of antigen (e.g. carbohydrates) are preferentially recognized by either low or high hydropathy score VLRB molecules. The loop length we observed in our study is consistent with findings from a recent report that used a short-read sequencing approach on RNA obtained from a single lamprey larva (*Lampetra planeri*) (47). The VLRB repertoire analysis of this dataset indicated a total VLRB repertoire of approximately 30,000 unique sequences, including those from tissue-resident VLRB cells. The substantially larger repertoires observed in our study might reflect differences in repertoire size between distinct lamprey species; alternatively, it supports the suitability of the long-read PacBio sequencing platform for VLRB repertoire analyses.

We were intrigued to observe identical fully assembled VLRB sequences isolated from distinct lamprey larvae. While identical sequences were most frequently observed among two different animals, we also detected identical VLRB sequences that were shared between all six larvae, even if at greatly reduced frequency, reminiscent of public antibody responses of jawed vertebrates. The concept of public antibody responses, defined as clonotypes with shared genetic elements from distinct donors, gained prominence over the last 10 years; it is implicated in the development of effective vaccines and was studied in immune responses to various infectious disease pathogens (48–54). Our finding of identical, fully mature VLRB sequences in distinct larvae is going beyond the characteristic of only shared genetic elements such as variable gene usage and complementarity determining region sequence similarity commonly used for public antibody responses in humans (51, 52, 55–57). In this context it is interesting to note that immunizations of sea lamprey larvae with influenza virus preparations revealed VLRB responses targeting preferentially hemagglutinin, comparable with antibody responses observed in jawed vertebrates (58).

Our analysis of bulk transcript analysis demonstrated the feasibility of the PacBio platform to obtain large scale VLRB sequence data. It will be interesting to expand this approach to single cell level data analysis, which in turn would permit a comparative analysis of the clonal composition of jawless and jawed vertebrate immune repertoires and facilitate the monitoring of antigen specific clones following immunizations with defined antigens. Our cluster analysis of the VLRB repertoires of all 6 larvae revealed several considerably expanded sequence clusters. While it is tempting to interpret these clusters as an indication of clonal expansion in response to an ongoing pathogenic challenge, formal demonstration of clonal expansion will require immune repertoire analysis on a single cell level. It is also important to take into account that the immune repertoire analysis performed in this study was limited to circulating VLRB cells. Tissue based cells of the adaptive immune system of jawed vertebrates, especially those found in mucosal tissues perform key tasks in the protection from pathogenic challenges and tissue based VLRB cells will have to be included in a detailed comparative analysis of the immune repertoires and clonal distribution of jawless and jawed vertebrates. Nonetheless, establishing extensive VLRB repertoire databases is the first step for the development of machine learning strategies to rapidly isolate VLRB sequences with desired antigen specificity and to subsequently explore these reagents for potential biomedical research as well as clinical applications.

## Data availability statement

The data presented in the study are deposited in the Sequence Read Archive (SRA), accession number SRR29656293.

## Ethics statement

The animal study was approved by Animal Care Committee, Research Oversight & Compliance Division of the Vice-President, Research & Innovation University of Toronto. The study was conducted in accordance with the local legislation and institutional requirements.

## Author contributions

AZ: Conceptualization, Data curation, Formal analysis, Investigation, Methodology, Resources, Software, Validation, Visualization, Writing – original draft, Writing – review & editing. AO: Writing – review & editing. IF: Writing – review & editing. AT: Writing – review & editing. AV: Writing – review & editing. SD: Writing – review & editing. LL: Writing – review & editing. VD: Writing – review & editing. OO: Writing – review & editing. GE: Conceptualization, Formal analysis, Funding acquisition, Investigation, Methodology, Project administration, Resources, Supervision, Validation, Visualization, Writing – original draft, Writing – review & editing.

## References

[1] Tonegawa S. Somatic generation of antibody diversity. Nature. (1983) 302:575–81. doi: 10.1038/302575a0 6300689

[2] Rajewsky K. Clonal selection and learning in the antibody system. Nature. (1996) 381:751–8. doi: 10.1038/381751a0 8657279

[3] Victora GD Nussenzweig MC. Germinal centers. Annu Rev Immunol. (2022) 40:413–42. doi: 10.1146/annurev-immunol-120419-022408 35113731

[4] Mouquet H Scheid JF Zoller MJ Krogsgaard M Ott RG Shukair S. Polyreactivity increases the apparent affinity of anti-Hiv antibodies by heteroligation. Nature. (2010) 467:591–5. doi: 10.1038/nature09385 PMC3699875 20882016

[5] Smith K Crowe SR Garman L Guthridge CJ Muther JJ McKee E. Human monoclonal antibodies generated following vaccination with Ava provide neutralization by blocking furin cleavage but not by preventing oligomerization. Vaccine. (2012) 30:4276–83. doi: 10.1016/j.vaccine.2012.03.002 PMC3367042 22425791

[6] Wardemann H Yurasov S Schaefer A Young JW Meffre E Nussenzweig MC. Predominant autoantibody production by early human B cell precursors. Science. (2003) 301:1374–7. doi: 10.1126/science.1086907 12920303

[7] Duty JA Szodoray P Zheng N-Y Koelsch KA Zhang Q Swiatkowski M. Functional anergy in a subpopulation of naive B cells from healthy humans that express autoreactive immunoglobulin receptors. J Exp Med. (2009) 206:139–51. doi: 10.1084/jem.20080611 PMC2626668 19103878

[8] Samuels J Ng YS Coupillaud C Paget D Meffre E. Impaired early B cell tolerance in patients with rheumatoid arthritis. J Exp Med. (2005) 201:1659–67. doi: 10.1084/jem.20042321 PMC2212916 15897279

[9] Robbiani DF Gaebler C Muecksch F Lorenzi JCC Wang Z Cho A. Convergent antibody responses to Sars-Cov-2 in convalescent individuals. Nature. (2020) 584:437–42. doi: 10.1038/s41586-020-2456-9 PMC7442695 32555388

[10] Woodruff MC Ramonell RP Nguyen DC Cashman KS Saini AS Haddad NS. Extrafollicular B cell responses correlate with neutralizing antibodies and morbidity in Covid-19. Nat Immunol. (2020) 21:1506–16. doi: 10.1038/s41590-020-00814-z PMC7739702 33028979

[11] He B Liu S Wang Y Xu M Cai W Liu J. Rapid isolation and immune profiling of Sars-Cov-2 specific memory B cell in convalescent Covid-19 patients via Libra-Seq. Signal Transduct Target Ther. (2021) 6:195. doi: 10.1038/s41392-021-00610-7 34001847 PMC8127497

[12] Jin W Yang Q Peng Y Yan C Li Y Luo Z. Single-cell Rna-Seq reveals transcriptional heterogeneity and immune subtypes associated with disease activity in human myasthenia gravis. Cell Discovery. (2021) 7:85. doi: 10.1038/s41421-021-00314-w 34521820 PMC8440681

[13] Liu Y Budylowski P Dong S Li Z Goroshko S Leung LYT. Sars-Cov-2-reactive mucosal B cells in the upper respiratory tract of uninfected individuals. J Immunol. (2021) 207:2581–8. doi: 10.4049/jimmunol.2100606 34607939

[14] Natali EN Horst A Meier P Greiff V Nuvolone M Babrak LM. The dengue-specific immune response and antibody identification with machine learning. NPJ Vaccines. (2024) 9:16. doi: 10.1038/s41541-023-00788-7 38245547 PMC10799860

[15] Natali EN Babrak LM Miho E. Prospective artificial intelligence to dissect the dengue immune response and discover therapeutics. Front Immunol. (2021) 12:574411. doi: 10.3389/fimmu.2021.574411 34211454 PMC8239437

[16] Horst A Smakaj E Natali EN Tosoni D Babrak LM Meier P. Machine learning detects anti-Denv signatures in antibody repertoire sequences. Front Artif Intell. (2021) 4:715462. doi: 10.3389/frai.2021.715462 34708197 PMC8542978

[17] Pancer Z Amemiya CT Ehrhardt GRA Ceitlin J Gartland GL Cooper MD. Somatic diversification of variable lymphocyte receptors in the Agnathan Sea Lamprey. Nature. (2004) 430:174–80. doi: 10.1038/nature02740 15241406

[18] Boehm T McCurley N Sutoh Y Schorpp M Kasahara M Cooper MD. Vlr-based adaptive immunity. Annu Rev Immunol. (2012) 30:203–20. doi: 10.1146/annurev-immunol-020711-075038 PMC3526378 22224775

[19] Boehm T Hirano M Holland SJ Das S Schorpp M Cooper MD. Evolution of alternative adaptive immune systems in vertebrates. Annu Rev Immunol. (2018) 36:19–42. doi: 10.1146/annurev-immunol-042617-053028 29144837

[20] Herrin BR Alder MN Roux KH Sina C Ehrhardt GRA Boydston JA. Structure and specificity of lamprey monoclonal antibodies. Proc Natl Acad Sci U.S.A. (2008) 105:2040–5. doi: 10.1073/pnas.0711619105 PMC2542867 18238899

[21] Guo P Hirano M Herrin BR Li J Yu C Sadlonova A. Dual nature of the adaptive immune system in lampreys. Nature. (2009) 459:796–801. doi: 10.1038/nature08068 19474790 PMC2714547

[22] Hirano M Guo P McCurley N Schorpp M Das S Boehm T. Evolutionary implications of a third lymphocyte lineage in lampreys. Nature. (2013) 501:435–8. doi: 10.1038/nature12467 PMC3901013 23934109

[23] Das S Boehm T Holland SJ Rast JP Fontenla-Iglesias F Morimoto R. Evolution of two distinct variable lymphocyte receptors in lampreys: Vlrd and Vlre. Cell Rep. (2023) 42:112933. doi: 10.1016/j.celrep.2023.112933 37542721 PMC11160967

[24] Alder MN Rogozin IB Iyer LM Glazko GV Cooper MD Pancer Z. Diversity and function of adaptive immune receptors in a jawless vertebrate. Science. (2005) 310:1970–3. doi: 10.1126/science.1119420 16373579

[25] Rogozin IB Iyer LM Liang L Glazko GV Liston VG Pavlov YI. Evolution and diversification of lamprey antigen receptors: evidence for involvement of an Aid-Apobec family cytosine deaminase. Nat Immunol. (2007) 8:647–56. doi: 10.1038/ni1463 17468760

[26] Konning D Zielonka S Grzeschik J Empting M Valldorf B Krah S. Camelid and shark single domain antibodies: structural features and therapeutic potential. Curr Opin Struct Biol. (2017) 45:10–6. doi: 10.1016/j.sbi.2016.10.019 27865111

[27] Han BW Herrin BR Cooper MD Wilson IA. Antigen recognition by variable lymphocyte receptors. Science. (2008) 321:1834–7. doi: 10.1126/science.1162484 PMC2581502 18818359

[28] Velikovsky CA Deng L Tasumi S Iyer LM Kerzic MC Aravind L. Structure of a lamprey variable lymphocyte receptor in complex with a protein antigen. Nat Struct Mol Biol. (2009) 16:725–30. doi: 10.1038/nsmb.1619 PMC2722044 19543291

[29] McKitrick TR Bernard SM Noll AJ Collins BC Goth CK McQuillan AM. Novel lamprey antibody recognizes terminal sulfated galactose epitopes on mammalian glycoproteins. Commun Biol. (2021) 4:674. doi: 10.1038/s42003-021-02199-7 34083726 PMC8175384

[30] Chan JTH Liu Y Khan S St-Germain JR Zou C Leung LYT. A tyrosine sulfation-dependent Hla-I modification identifies memory B cells and plasma cells. Sci Adv. (2018) 4:eaar7653. doi: 10.1126/sciadv.aar7653 30417091 PMC6221509

[31] Yu C Liu Y Chan JTH Tong J Li Z Shi M. Identification of human plasma cells with a lamprey monoclonal antibody. JCI Insight. (2016) 1. doi: 10.1172/jci.insight.84738 PMC4854299 27152361

[32] Lajoie JM Katt ME Waters EA Herrin BR Shusta EV. Identification of lamprey variable lymphocyte receptors that target the brain vasculature. Sci Rep. (2022) 12:6044. doi: 10.1038/s41598-022-09962-8 35411012 PMC9001667

[33] Umlauf BJ Clark PA Lajoie JM Georgieva JV Bremner S Herrin BR. Identification of variable lymphocyte receptors that can target therapeutics to pathologically exposed brain extracellular matrix. Sci Adv. (2019) 5:eaau4245. doi: 10.1126/sciadv.aau4245 31106264 PMC6520025

[34] Wenger AM Peluso P Rowell WJ Chang P-C Hall RJ Concepcion GT. Accurate circular consensus long-read sequencing improves variant detection and assembly of a human genome. Nat Biotechnol. (2019) 37:1155–62. doi: 10.1038/s41587-019-0217-9 PMC6776680 31406327

[35] Edgar RC. Search and clustering orders of magnitude faster than blast. Bioinformatics. (2010) 26:2460–1. doi: 10.1093/bioinformatics/btq461 20709691

[36] Chenna R Sugawara H Koike T Lopez R Gibson TJ Higgins DG. Multiple sequence alignment with the clustal series of programs. Nucleic Acids Res. (2003) 31:3497–500. doi: 10.1093/nar/gkg500 PMC168907 12824352

[37] Li W Godzik A. Cd-hit: A fast program for clustering and comparing large sets of protein or nucleotide sequences. Bioinformatics. (2006) 22:1658–9. doi: 10.1093/bioinformatics/btl158 16731699

[38] Fu L Niu B Zhu Z Wu S Li W. Cd-hit: accelerated for clustering the next-generation sequencing data. Bioinformatics. (2012) 28:3150–2. doi: 10.1093/bioinformatics/bts565 PMC3516142 23060610

[39] Gupta NT Vander Heiden JA Uduman M Gadala-Maria D Yaari G Kleinstein SH. Change-O: A toolkit for analyzing large-scale B cell immunoglobulin repertoire sequencing data. Bioinformatics. (2015) 31:3356–8. doi: 10.1093/bioinformatics/btv359 PMC4793929 26069265

[40] Das S Rast JP Li J Kadota M Donald JA Kuraku S. Evolution of variable lymphocyte receptor B antibody loci in jawless vertebrates. Proc Natl Acad Sci U.S.A. (2021) 118. doi: 10.1073/pnas.2116522118 PMC8685922 34880135

[41] Nagawa F Kishishita N Shimizu K Hirose S Miyoshi M Nezu J. Antigen-receptor genes of the Agnathan lamprey are assembled by a process involving copy choice. Nat Immunol. (2007) 8:206–13. doi: 10.1038/ni1419 17187071

[42] Kelley LA Mezulis S Yates CM Wass MN Sternberg MJ. The Phyre2 web portal for protein modeling, prediction and analysis. Nat Protoc. (2015) 10:845–58. doi: 10.1038/nprot.2015.053 PMC5298202 25950237

[43] Crooks GE Hon G Chandonia J-M Brenner SE. Weblogo: A sequence logo generator. Genome Res. (2004) 14:1188–90. doi: 10.1101/gr.849004 PMC419797 15173120

[44] Gunn RJ Herrin BR Acharya S Cooper MD Wilson IA. Vlr recognition of Tlr5 expands the molecular characterization of protein antigen binding by non-Ig-based antibodies. J Mol Biol. (2018) 430:1350–67. doi: 10.1016/j.jmb.2018.03.016 PMC5915902 29596914

[45] Collins BC Gunn RJ McKitrick TR Cummings RD Cooper MD Herrin BR. Structural insights into Vlr fine specificity for blood group carbohydrates. Structure. (2017) 25:1667–78.e4. doi: 10.1016/j.str.2017.09.003 28988747 PMC5677568

[46] Ardavin CF Zapata A. Ultrastructure and changes during metamorphosis of the lympho-hemopoietic tissue of the larval anadromous sea lamprey petromyzon marinus. Dev Comp Immunol. (1987) 11:79–93. doi: 10.1016/0145-305X(87)90010-3 3595947

[47] Morimoto R O’Meara CP Holland SJ Trancoso I Souissi A Schorpp M. Cytidine deaminase 2 is required for Vlrb antibody gene assembly in lampreys. Sci Immunol. (2020) 5. doi: 10.1126/sciimmunol.aba0925 32169953

[48] Andrews SF McDermott AB. Shaping a universally broad antibody response to influenza amidst a variable immunoglobulin landscape. Curr Opin Immunol. (2018) 53:96–101. doi: 10.1016/j.coi.2018.04.009 29730560

[49] Lanzavecchia A Fruhwirth A Perez L Corti D. Antibody-guided vaccine design: identification of protective epitopes. Curr Opin Immunol. (2016) 41:62–7. doi: 10.1016/j.coi.2016.06.001 27343848

[50] Parameswaran P Liu Y Roskin KM Jackson KKL Dixit VP Lee J-Y. Convergent antibody signatures in human dengue. Cell Host Microbe. (2013) 13:691–700. doi: 10.1016/j.chom.2013.05.008 23768493 PMC4136508

[51] Pieper K Tan J Piccoli L Foglierini M Barbieri S Chen Y. Public antibodies to malaria antigens generated by two Lair1 insertion modalities. Nature. (2017) 548:597–601. doi: 10.1038/nature23670 28847005 PMC5635981

[52] Setliff I McDonnell WJ Raju N Bombardi RG Murji AA Scheepers C. Multi-donor longitudinal antibody repertoire sequencing reveals the existence of public antibody clonotypes in Hiv-1 infection. Cell Host Microbe. (2018) 23:845–54.e6. doi: 10.1016/j.chom.2018.05.001 29861170 PMC6002606

[53] Wu NC Wilson IA. Structural insights into the design of novel anti-influenza therapies. Nat Struct Mol Biol. (2018) 25:115–21. doi: 10.1038/s41594-018-0025-9 PMC5930012 29396418

[54] Wang Y Yuan M Lv H Peng J Wilson IA Wu NC. A large-scale systematic survey reveals recurring molecular features of public antibody responses to Sars-Cov-2. Immunity. (2022) 55:1105–17 e4. doi: 10.1016/j.immuni.2022.03.019 35397794 PMC8947961

[55] Henry Dunand CJ Wilson PC. Restricted, canonical, stereotyped and convergent immunoglobulin responses. Philos Trans R Soc Lond B Biol Sci. (2015) 370. doi: 10.1098/rstb.2014.0238 PMC4528415 26194752

[56] Jackson KJL Liu Y Roskin KM Glanville J Hoh RA Seo K. Human responses to influenza vaccination show seroconversion signatures and convergent antibody rearrangements. Cell Host Microbe. (2014) 16:105–14. doi: 10.1016/j.chom.2014.05.013 PMC4158033 24981332

[57] Truck J Ramasamy MN Galson JD Rance R Parkhill J Lunter G. Identification of antigen-specific B cell receptor sequences using public repertoire analysis. J Immunol. (2015) 194:252–61. doi: 10.4049/jimmunol.1401405 PMC4272858 25392534

[58] Altman MO Bennink JR Yewdell JW Herrin BR. Lamprey Vlrb response to influenza virus supports universal rules of immunogenicity and antigenicity. Elife. (2015) 4. doi: 10.7554/eLife.07467 PMC4552221 26252514

