# Supplementary material for: High throughput long-read sequencing of circulating lymphocytes of the evolutionarily distant sea lamprey reveals diversity and common elements of the variable lymphocyte receptor B (VLRB) repertoire
Source: Front Immunol. 2024 Aug 7;15:1427075. doi: 10.3389/fimmu.2024.1427075 (PMC11335541; doi:10.3389/fimmu.2024.1427075)
Supplement: Supplementary file 1 [file DataSheet_1.pdf]

Supplementary Material

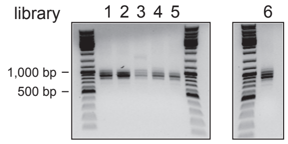

Supplementary Figure S1: PCR amplified VLRB sequences. cDNA was generated on total RNA from circulating lymphocytes of 6 individual sea lamprey larvae using UMI containing oligos as described. 10% of each of the purified pcr samples was analyzed by DNA electrophoresis using 1% agarose gels, the remainder of the samples were utilized for PacBio sequencing.

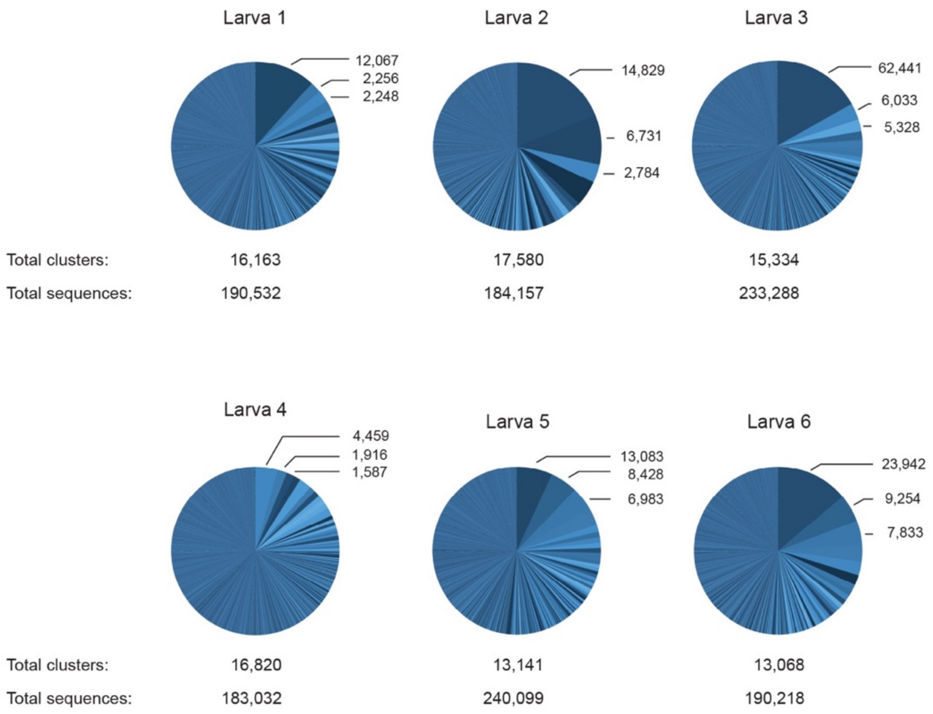

Supplementary Figure S2: Cluster analysis of VLRB sequences identified in each larval data set. Total cluster and total sequence counts are indicated. The sequence counts for the 3 largest clusters of each data set are indicated.

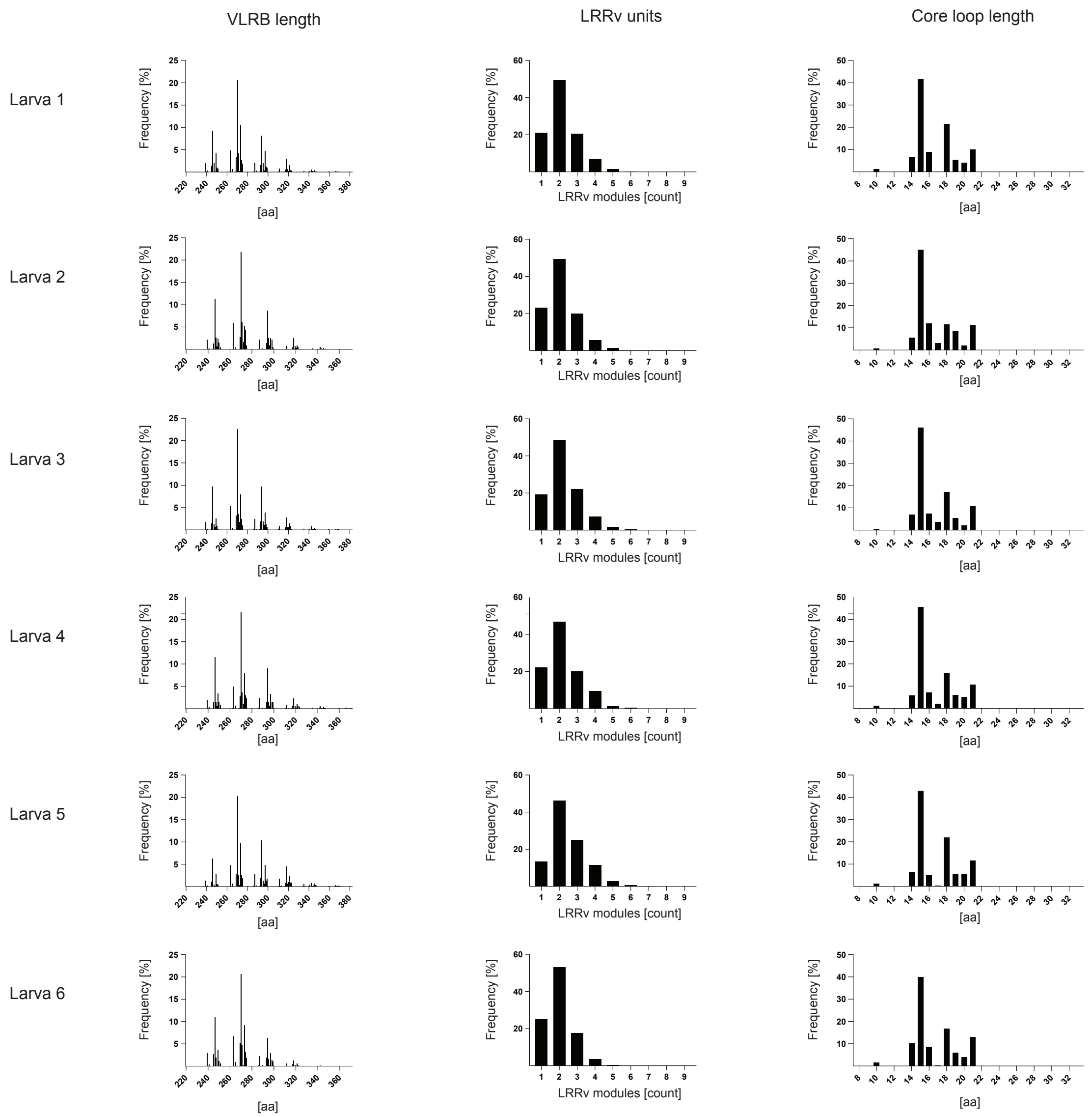

Supplementary Figure S3: Structural elements of VLRB molecules for individual sea lamprey larvae. Shown are the frequencies of overall VLRB length (left column), numbeers of LRRv units incorporated into VLRB molecules (center column), and length of core loop sequences detected in individual VLRB molecules. Frequencies were calculated relative to the total number of VLRB sequences for each individual VLRB larva dataset.

Subunit	Complete VLRB	SP	LRR-NT	LRR1	LRRv/LRRve	CP	LRR-CT	Loop (*)	stalk
**Length (aa)**	239-438	21	31	18	24	11	46-58	8-33	87

Supplementary Table 1: Structural components of consensus VLRB sequences. Shown is the length of the various components of consensus VLRB sequences defined for the VLRB repertoire sequence analysis. (*) Loop length refers to the core loop length and is included in the aa indicated for LRR-CT.
